# Hemostatic and thromboelastographic parameters in dogs with renal azotemia

**DOI:** 10.14202/vetworld.2023.1214-1221

**Published:** 2023-06-05

**Authors:** Hendryk Fischer, Vera Geisen, Roswitha Dorsch, Katrin Hartmann, René Dörfelt

**Affiliations:** LMU Small Animal Clinic, LMU Munich, Veterinaerstrasse 13, 80539 Munich, Germany

**Keywords:** acute kidney injury, canine, chronic kidney disease, coagulation, platelet function, viscoelastic test

## Abstract

**Background and Aim:**

Humans and dogs with azotemia can develop coagulation disorders. Therefore, this study aimed to evaluate the coagulation profiles and thromboelastographic parameters in dogs with acute kidney injury (AKI) and chronic kidney disease (CKD).

**Materials and Methods:**

In this prospective study, 31 client-owned dogs with renal azotemia (creatinine >220 μmol/L) were enrolled. Clinical signs of hemostatic disorders, complete blood count, coagulation profile, D-dimers, thromboelastography, and 28-day survival data were obtained and analyzed using the t-test, Mann–Whitney U test, and Chi-square test. Statistical significance was set at p < 0.05.

**Results:**

Seventeen dogs with AKI, 10 with CKD, and four with acute-on-chronic kidney injury (AoC) were enrolled. Ten dogs (AKI, 8/17; CKD, 2/10) had thrombocytopenia. Prothrombin time was prolonged in four dogs with AKI and longer in dogs with AKI than in dogs with CKD (p = 0.004). The activated partial thromboplastin time was prolonged in 23 dogs (AKI, 14/17; CKD, 7/10; AoC, 3/4) and was longer in azotemic dogs than in healthy control dogs (p = 0.003). Thromboelastographic tracings were hypocoagulable in three dogs with AKI and hypercoagulable in 16 dogs (AKI 4/17, CKD 9/10, AoC 3/4). The thromboelastographic values for maximum amplitude (p < 0.001) and global clot strength (p < 0.001) were lower in dogs with AKI than in those with CKD.

**Conclusion:**

Hypercoagulable thromboelastographic tracings were observed in dogs with CKD, whereas coagulation times were prolonged in dogs with AKI. However these findings should be validated by further studies.

## Introduction

The loss of excretory renal function leads to disturbances in the excretion of uremic toxins and can cause acid-base, electrolyte, and endocrine disorders. In acute kidney injury (AKI), kidney function and glomerular filtration rate (GFR) decrease abruptly, but are potentially reversible within hours to days [1]. The most common causes of AKI in dogs are hemodynamic disturbances, infectious diseases, and nephrotoxic substances [2]. In chronic kidney disease (CKD), azotemia and GFR worsen gradually and irreversibly over a period of 3 months or longer. Chronic kidney disease in dogs is rarely hereditary. In most cases, it is an acquired condition. The causes of CKD include glomerular diseases, infections, repeated ischemic events, nephrotoxicity, neoplasia, previous AKI, or urinary obstruction. Commonly, the cause of chronic changes leading to the loss of functional capacity cannot be determined [1, 3].

Azotemia is associated with coagulation disturbances in human patients with kidney diseases, leading to a 2-fold increased risk of bleeding [4]. In contrast, the risk of venous thrombosis in humans with CKD is increased 1.2–1.6 times compared with patients without kidney diseases [5–7]. The incidence of fatal pulmonary embolism in patients with kidney disease is 6.6%, which is almost 6-fold higher compared to patients without kidney disease [8]. Dogs with leptospirosis commonly suffer from azotemia, thrombocytopenia, and normo-, hypo-, and hypercoagulable states have been observed [9].

Viscoelastic tests are the only suitable tools for assessing hypercoagulable states. They provide a global overview of coagulation from initiation to fibrinolysis. The two most common viscoelastic tests are thromboelastography (TEG), and rotational thromboelastometry (ROTEM). Over the last three decades, viscoelastic testing has become increasingly popular as a point-of-care test, particularly in intensive care units and emergency rooms. To improve the comparability and use of viscoelastic testing in veterinary medicine, the first evidence-based guidelines on using viscoelastic testing in veterinary medicine (PROVETS) were published in 2014 [10]. Thus, viscoelastic testing is considered the gold standard for diagnosing platelet function and hypercoagulability in veterinary medicine.

This study aimed to evaluate primary, secondary, and tertiary hemostasis in azotemic dogs using viscoelastic testing, compare them to those in healthy dogs, and examine the association between changes in hemostasis and mortality. In addition, the differences in hemostatic parameters between dogs with CKD and those with AKI were investigated.

## Materials and Methods

### Ethical approval and Informed consent

The protocol for this prospective and clinical study was approved by the Ethics Committee of the Center of Clinical Veterinary Medicine (57-10-20-2015). Informed consent was obtained from all patients before enrolment.

### Study period and location

The study was conducted from November 2015 to July 2017 at LMU Small Animal Clinic, Munich.

### Animals

Thirty-one dogs with renal azotemia were included if their serum creatinine was >220 μmol/L, corresponding to International Renal Interest Society (IRIS) grade or stage ≥ III. To exclude pre-renal azotemia, urine-specific gravity was evaluated. Patients with pre-renal or post-renal azotemia were excluded from the study. Azotemia was classified as pre-renal in origin if patients had a urine-specific gravity ≥1030 and in patients with a urine-specific gravity <1030 that were dehydrated, had another non-renal disease that interfered with the urine concentrating capacity, or received medication that prohibited adequate urine concentration. Post-renal azotemia was excluded based on the absence of any clinical signs of urethral or ureteral obstruction and abdominal ultrasound findings of ureteral obstruction and urine leakage into the abdomen. The dogs receiving medications such as anticoagulants or platelet aggregation inhibitors within 14 days before presentation were excluded from the study.

In total, 31 dogs (ten intact males, five castrated males, nine intact females, and seven castrated females) with renal azotemia were included in the study. The breeds included five Golden Retrievers, three Labradors, three mixed-breed dogs, and other breeds with <4 animals per breed. Dogs were, on average, 7.4 ± 3.8 years old, with a median weight of 25.1 kg (4.3–53.2 kg).

Healthy control dogs consisted of ten client-owned, non-azotemic dogs that were part of an in-house blood donation program. Health status was determined based on medical history, physical examination, complete blood count, and serum chemistry analysis. The control group included three intact males, two castrated males, two intact females, and three castrated female dogs, with three Labradors and other breeds with < 3 animals per breed. The mean age (6.7 ± 3.2 years) and median weight (25.5 kg; 17.6–61.4 kg) did not differ significantly between azotemic dogs and the control group (p = 0.601; p = 0.339).

### Data collection

Medical history focused on clinical signs indicative of disturbed hemostasis, such as epistaxis, hematuria, hematemesis, or melena, as well as known medical conditions and current medications. A complete physical examination was performed. Abdominal ultrasonography was performed using a Diplomate ECVIM-CA (Logiq P6, GE Healthcare, Chalfont St. Giles, United Kingdom).

At presentation, a complete blood count, including platelet count (Sysmex XT-2000i, Sysmex Corporation, Kobe, Japan), serum chemistry (COBAS INTEGRA 400 plus, F. Hoffmann-La Roche AG, Basel, Switzerland), and blood gas analysis (RAPIDPoint 450, Siemens AG, Berlin, Germany) were performed in all dogs. Urinalysis was also performed in all dogs and included measurement of urine-specific gravity, test stick analysis (Combur 5 Sticks, F. Hoffmann-La Roche AG), urine sediment, urine protein/creatinine ratio in the case of inactive sediment (COBAS INTEGRA 400 plus, F. Hoffmann-La Roche AG), and bacterial culture. In addition, coagulation tests (activated partial thromboplastin time [aPTT]; prothrombin time [PT]; CL4, KG Behnk Elektronik GmbH & Co., Norderstedt, Germany), D-dimer analysis (Nyco-Card Reader II, Alere Inc., Waltham, USA), and kaolin-activated thromboelastograph (TEG 5000, Haemonetics S.A. Signy, Switzerland) were performed at presentation. Blood samples for coagulation and TEG were obtained, handled, and analyzed according to established guidelines [10]. The TEG parameters of reaction time, kinetics, alpha angle, maximum amplitude (MA), global clot strength (G), clot lysis at 30 min (Ly30), and clot lysis at 60 min (Ly60) were analyzed.

G was calculated as follows:

G = 5000 × MA/100 – MA.

Hypercoagulable TEG tracings were defined as increased and hypocoagulable TEG tracings were defined as decreased G (reference range, 3200–9600 dynes/cm^2^). Hyperfibrinolysis was defined as increased Ly30 (reference range, 0%–8%) or Ly60 (reference range, 0%–15%).

### Classification of dogs

**Figure-1:**
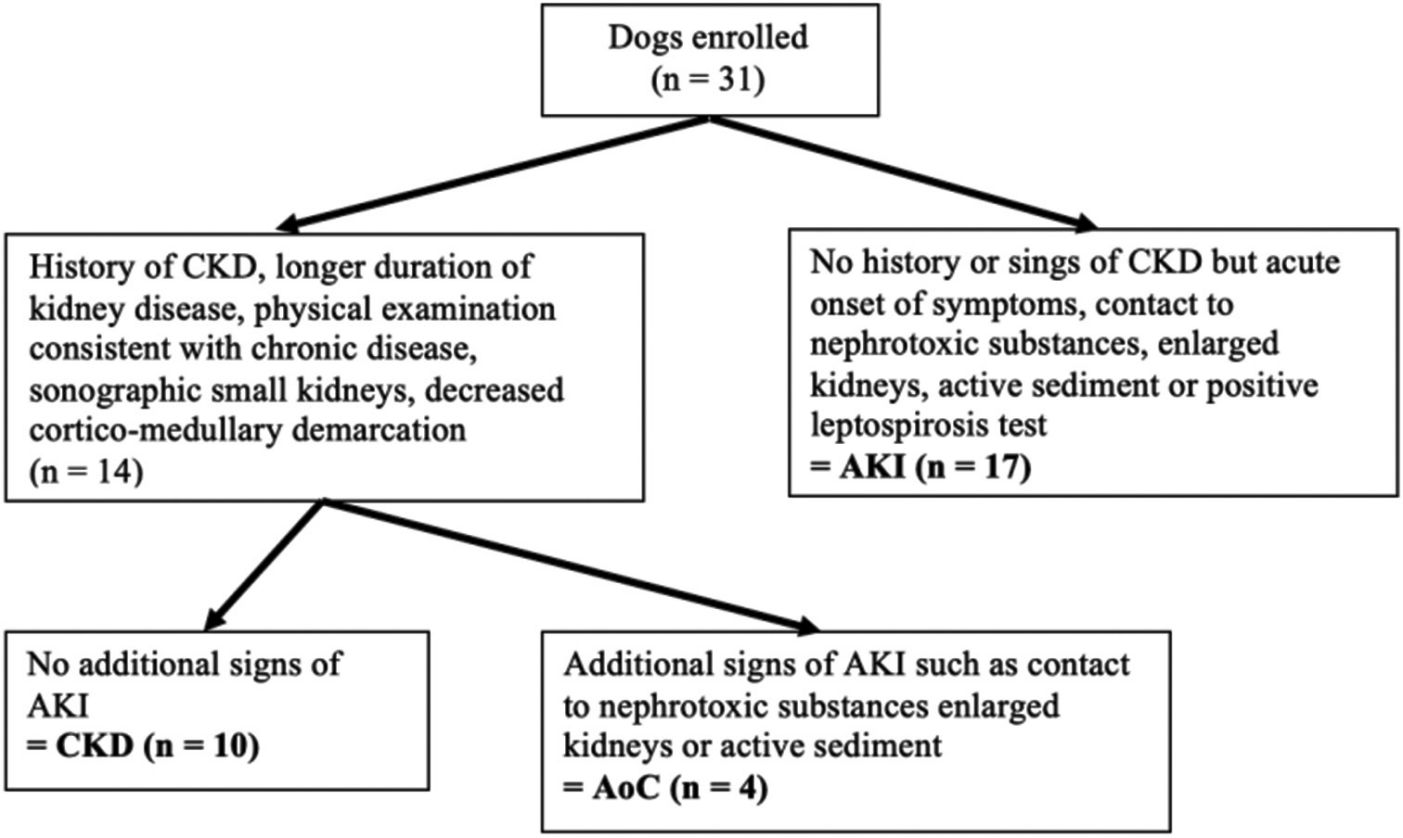
Classification of 31 azotemic dogs according to history clinical examination, urine analysis, and sonography. AKI=Acute kidney injury, CKD=Chronic kidney disease, AoC=Acute on chronic kidney disease.

Based on signalment, history, physical examination, sonographic findings, and urinalysis results, dogs were assigned to one of three study groups: AKI, CKD, or acute-on-chronic kidney injury (AoC). Acute kidney injury was defined as the absence of a history of chronic diseases or laboratory changes indicating renal damage, sudden onset of symptoms, and additional signs of AKI, such as active urinary sediment and sonographic normal to enlarged kidneys. Exposure to nephrotoxic substances, such as grapes, raisins, or ethylene glycol, or a positive test (urine polymerase chain reaction or serum titer pair with increasing titer within approximately 3 weeks) for leptospirosis further corroborated the diagnosis of AKI. Chronic kidney disease was defined as a longer (at least 3 months) duration of kidney disease, confirmed by previous laboratory findings or history, such as polyuria/polydipsia; physical examination findings consistent with chronic diseases, such as weight loss; or evidence of chronic sonographic abnormalities of the kidneys, such as small kidneys, renal infarcts, and decreased cortico-medullary differentiation [1]. The AoC group included dogs with CKD, but with evidence of an acute insult, such as active sediment or contact with nephrotoxic substances (Figure-1). Dogs with AoC were excluded from the comparison of parameters between the AKI and CKD groups. The cause of renal damage was determined and noted. Follow-up was performed 28 days later to determine short-term survival rates.

### Statistical analysis

Statistical analyses were performed using commercially available software (GraphPad Prism, 5.04; GraphPad Software Inc., San Diego, USA). Data were tested for normality using the D’Agostino Omnibus test. Normally distributed values are reported as mean ± standard deviation, whereas non-normally distributed values are reported as median and range.

Absolute coagulation and thromboelastographic parameters were analyzed using the Mann–Whitney U test and t-test. The Chi-square, Fisher’s exact, and Friedman tests were used to compare the number of patients with hyper- and hypocoagulable states. The correlation of creatinine, IRIS grade/stage, hematocrit with coagulation, and thromboelastographic parameters was analyzed, depending on normality, using Spearman correlation or Pearson correlation. Statistical significance was set at p < 0.05.

## Results

### Categorization of azotemic dogs

Of the 31 azotemic dogs, 17 were assigned to AKI, ten to CKD, and four to AoC (Figure-1). The causes of AKI were leptospirosis in four, grape intoxication in three, non-steroidal anti-inflammatory drug (NSAID) intoxication in two, and anesthesia-related following intestinal foreign body surgery in one dog, and unknown in seven dogs. The causes of CKD were unknown in seven, and congenital nephropathy, glomerulopathy, and previous NSAID intoxication were reported in one dog each. Two dogs with CKD had protein-losing nephropathy with urine-protein/creatinine ratio of 18.1 and 19.9, respectively, and albumin levels below 20 g/L. The cause of the acute component in the AoC group was unknown in three dogs and ascending urinary tract infection in one dog.

### Comparison of azotemic and healthy dogs

**Figure-2:**
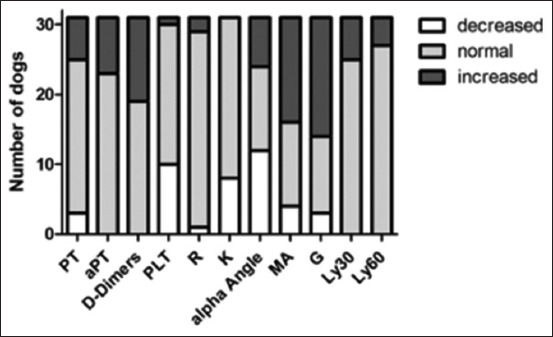
Coagulation times, platelet count, and thromboelastographic values of 31 azotemic dogs in relation to the reference values. aPTT=Activated partial thromboplastin time, Crea=creatinine, G=Global clot strength, K=Kinetics, Ly30=Clot lysis after 30 min, Ly60=Clot lysis after 60 min, MA=Maximum amplitude, Plt=Platelets, PT=Prothrombin time, R=Reaction time.

The clinical parameters of heart rate (p < 0.001) and respiratory rate (p = 0.009) were higher and body temperature was lower in azotemic dogs than in control dogs (p = 0.030; Table-1). Azotemic dogs more frequently had increased aPTT (23/31; control dogs 2/10; p = 0.008), decreased platelet count (10/31; control dogs 0/10; p = 0.043), increased maximum amplitude (MA) (14/31; control dogs 0/10; p = 0.005), and increased G (17/31; control dogs 0/10; p = 0.002) than control dogs (Figure-2). Azotemic dogs had significantly higher mean aPTT (p = 0.003), median MA (p = 0.028), and Ly30 (p = 0.022) than control dogs (Table-1).

**Table-1:** Demographic data, biochemical values, platelet count, coagulation times, and thromboelastographic values of 31 azotemic and 10 control dogs.

Parameter	RI	Control dogs (n = 10)	Azotemic dogs (n = 31)	p-value
Age (years)		6.7 ± 3.2	7.4 ± 3.8	0.601
Weight (kg)		25.5 (17.6–61.4)	25.1 (4.3–53.2)	0.339
IRIS stage/grade			4 (3–5)	
Respiratory rate (/min)		20 (12–80)	28 (16–120)	**0.009**
Heart rate (/min)		84 (60–104)	108 (68–200)	**<0.001**
Temperature (C°)		38.6 (38.0–39.0)	38.1 (36.1–40.8)	**0.030**
Urea (mmol/L)	3.5–10.8	5.8 ± 2.2	52.5 ± 24.5	**<0.001**
Crea (µmol/L)	44–125	88 ± 18	691 ± 366	**<0.001**
Plt (G/L)	150–500	220 ± 50	237 ± 138	0.713
PT (s)	13.8–23.2	18.5 (12.3–22.5)	18.3 (12.4–32.5)	0.988
aPTT (s)	10.0–13.2	12.5 ± 1.4	14.9 ± 2.2	**0.003**
D-Dimer	< 200	100 (90–400)	200 (90–2,800)	0.169
R (min)	1.8–8.6	4.0 ± 1.4	4.7 ± 1.8	0.313
K (min)	1.3–5.7	2.4 (1.4–12.0)	1.6 (0.8–5.1)	0.053
Alpha angle (°)	36.9–74.6	60.3 (19.6–70.7)	65.7 (31.6–80.0)	0.081
MA (mm)	42.9–67.9	56.5 (33.5–66.1)	67.4 (31.0–82.7)	**0.028**
Ly30 (%)	0–8	0.0 (0.0–5.0)	1.0 (0.0–47.0)	**0.022**
Ly60 (%)	0–15	0.1 (0.0–8.6)	2.5 (1.0–56.7)	0.131
G (dynes/cm^2^)	3.200–9.600	6.484 ± 1.975	10.214 ± 5.852	0.056

Normally distributed data are presented as mean ± standard deviation, and were analyzed using the t-test. Not normally distributed data are presented as median (range) and were analyzed using the Mann–Whitney U test. aPTT=Activated partial thromboplastin time, Crea=Creatinine, G=Global clot strength, K=Kinetics, Ly30=Clot lysis after 30 min, Ly60=Clot lysis after 60 min, MA=Maximum amplitude, p-value=significance level, Plt=Platelets, PT=Prothrombin time, R=Reaction time, RI=Reference interval. Bold numbers indicate statistical significance

Prothrombin time was moderately correlated with IRIS grade and stage. None of the other hemostatic parameters correlated with creatinine or IRIS grade or stage (Table-2).

**Table-2:** Correlation of hemostatic parameter with creatinine and IRIS grade/stage in 31 azotemic dogs.

Parameter	Correlation with creatinine (Pearson)		Correlation with IRIS grade/stage (Spearmann)	
	
	r	p-value	R	p-value
Plt (G/L)	−0.055	0.769	0.016	0.932
PT (s)	0.288	0.117	**0.500**	**0.004**
aPTT (s)	0.086	0.647	0.054	0.7726
D-dimers	0.187	0.314	−0.072	0.702
R (min)	−0.074	0.691	0.016	0.933
K (min)	−0.073	0.696	0.045	0.811
Alpha angel (°)	0.090	0.630	0.024	0.898
MA (mm)	0.058	0.755	−0.047	0.804
Ly 30 (%)	−0.111	0.551	−0.114	0.542
Ly 60 (%)	−0.200	0.281	−0.192	0.302
G (dyn/cm^2^)	−0.061	0.743	−0.047	0.804

aPTT=Activated partial thromboplastin time, G=Global clot strength, K=Kinetics, Ly30=Clot lysis after 30 min, Ly60=Clot lysis after 60 min, MA=Maximum amplitude, p-value=Significance level, Plt=Platelets, PT=Prothrombin time, r=Pearson’s correlation coefficient, R=Spearman’s correlation coefficient, R=Reaction time. Bold numbers indicate statistical significance

### Outcome of azotemic dogs

Thirteen azotemic dogs were still alive 28 days after initial presentation, whereas 18 dogs died or were euthanized. Surviving dogs had lower urea levels (p = 0.024). Increases in aPTT (7/13) and D-dimer levels (2/13) were less frequently observed in surviving dogs than in non-survivors (aPTT: 16/18; p = 0.043; D-dimer 10/18; p = 0.032). Hypercoagulable TEG tracings were observed in 7/13 surviving and 10/18 non-surviving dogs (p = 1.000). Absolute coagulation times, platelet counts, and TEG parameters did not differ between survivors and non-survivors (Table-3).

**Table-3:** Biochemical values, platelet count, coagulation times, and thromboelastographic values of 13 surviving and 18 non-surviving azotemic dogs.

Parameter	RI	Survivor (n = 13)	Non-survivor (n = 18)	p-value
Urea (mmol/L)	3.5–10.8	41.0 ± 23.2	60.8 ± 22.6	**0.024**
Crea (µmol/L)	44–125	615 ± 337	746 ± 386	0.332
Plt (G/L)	150–500	236 ± 126	238 ± 149	0.970
PT (s)	13.8–23.2	18.3 (12.2–32.5)	18.2 (13.2–31.1)	0.522
aPTT (s)	10.0–13.2	14.5 ± 2.5	15.1 ± 1.9	0.402
D-Dimers	<200	100 (90–600)	300 (90–2.800)	0.056
R (min)	1.8–8.6	4.9 ± 2.1	4.5 ± 1.7	0.593
K (min)	1.3–5.7	1.8 (0.8–5.1)	1.5 (0.8–3.9)	0.457
Alpha angle (°)	36.9–74.6	65.7 (31.6–77.7)	67.2 (45.1–80.0)	0.704
MA (mm)	42.9–67.9	66.6 (37.9–81.0)	68.7 (31.0–82.7)	0.920
Ly30 (%)	0–8	0.0 (0.0–47.0)	4.1 (0.0–12.2)	0.205
Ly60 (%)	0–15	0.0 (0.0–56.7)	6.4 (0.0–40.3)	0.103
G (dynes/cm^2^)	3.200–9.600	9.970 (3.052–21.316)	10.950 (2.246–23.902)	0.920

Normally distributed data are presented as mean ± standard deviation and were analyzed using the t-test. Not normally distributed data are presented as median (range) and were analyzed using the Mann–Whitney U test. aPTT=Activated partial thromboplastin time, Crea=Creatinine, G=Global clot strength, K=Kinetics, Ly30=Clot lysis after 30 min, Ly60=Clot lysis after 60 min, MA=Maximum amplitude, p-value=Significance level, Plt=Platelets, PT=Prothrombin time, R=Reaction time, RI=Reference interval. Bold numbers indicate statistical significance

### Comparison of dogs with AKI to dogs with CKD

**Figure-3:**
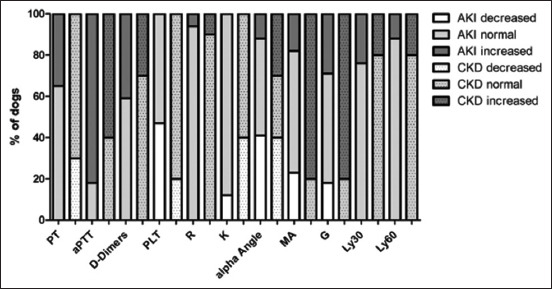
Coagulation times, platelet count, and thromboelastographic values of 17 dogs with AKI and 10 dogs with CKD in relation to the reference values. aPTT=Activated partial thromboplastin time, Crea=Creatinine, G=Global clot strength, K=Kinetics, Ly30=Clot lysis after 30 min, Ly60=Clot lysis after 60 min, MA=Maximum amplitude, Plt=Platelets, PT=Prothrombin time, R=Reaction time, AKI=Acute kidney injury, CKD=Chronic kidney disease.

Prothrombin time was prolonged in 14/17 dogs with AKI and in 7/10 dogs with CKD (p = 0.013). Thrombocytopenia was observed in 8/17 dogs with AKI and 2/10 dogs with CKD (p = 0.223). The MA and G were more frequently increased in dogs with CKD (MA: 8/10; G: 8/10) than in dogs with AKI (MA: 3/17; p = 0.012; G: 5/17; p = 0.018; Figure-3). Hypocoagulable TEG was observed in three dogs with AKI, with one of these dogs also being thrombocytopenic. Hypercoagulable TEG was present in 5/17 dogs with AKI, and in 8/10 dogs with CKD (p = 0.018). Hyperfibrinolytic TEG was observed in five dogs with AKI and two dogs with CKD (p = 0.678).

The mean MA and G were lower in dogs with AKI than those with CKD (p < 0.001 and p < 0.001, respectively). Mean PT was higher in dogs with AKI than in those with CKD (p = 0.004; Table-4).

**Table-4:** Biochemical values, platelet count, coagulation times, and thromboelastographic values of 17 azotemic dogs with AKI and 10 with CKD.

Parameter	RI	AKI (n = 17)	CKD (n = 10)	p-value
Urea (mmol/L)	3.5–10.8	56.5 ± 24.0	43.7 ± 24.4	0.197
Crea (µmol/L)	44–125	783 ± 418	544 ± 300	0.127
Plt (G/L)	150–500	196 ± 124	272 ± 144	0.157
PT (s)	13.8–23.2	22.1 ± 6.0	15.7 ± 2.7	0.004
aPTT (s)	10.0–13.2	15.6 ± 2.3	14.1 ± 2.0	0.084
D-Dimers	<200	200 (90–900)	150 (90–2,800)	0.721
R (min)	1.8–8.6	4.1 (3.1–9.3)	5.1 (1.9–8.7)	0.339
K (min)	1.3–5.7	1.8 (0.9–5.1)	1.5 (0.8–2.8)	0.217
Alpha angle (°)	36.9–74.6	61.5 ± 12.1	68.2 ± 9.0	0.145
MA (mm)	42.9–67.9	55.1 ± 12.5	72.9 ± 7.4	<0.001
Ly30 (%)	0–8	2.3 (0.0–47.0)	0.6 (0.0–11.8)	0.937
Ly60 (%)	0–15	2.9 (0.0–56.7)	1.9 (0–18.9)	0.697
G (dynes/cm^2^)	3.200–9.600	6.931 ± 3.092	14.815 ± 5.583	<0.001

Normally distributed data are presented as mean ± standard deviation and were analyzed using the t-test. Non-normally distributed data are presented as median (range) and were analyzed using the Mann–Whitney U test. aPTT=Activated partial thromboplastin time, Crea=Creatinine, G=Global clot strength, K=Kinetics, Ly30=Clot lysis after 30 min, Ly60=Clot lysis after 60 min, MA=Maximum amplitude, p-value=Significance level, Plt=Platelets, PT=Prothrombin time, R=Reaction time, RI=Reference interval. Bold numbers indicate statistical significance

### Clinical signs of hemorrhage

Clinical signs of hemorrhage, including melena (n = 3), petechia (n = 1), and hematemesis (n = 1), were observed in five dogs. Four of these dogs had AKI and one had CKD. Two additional dogs with hemorrhage due to a non-renal, non-coagulopathic process (melena after enterectomy [n = 1] and hematuria due to bacterial cystitis [n = 1]) were excluded from the analysis. No significant differences were observed between azotemic dogs with and without clinical signs of decreased hemostasis concerning coagulation and thromboelastographic parameters (Table-5). Two of the five dogs with signs of hemorrhage survived, while 10/24 dogs without signs of hemorrhage survived (p = 0.453).

**Table-5:** Biochemical values, platelet count, coagulation times, and thromboelastographic values in five azotemic dogs with, and 24 azotemic dogs without clinical signs of hemorrhage.

Parameter	RI	Hemorrhage (n = 5)	No hemorrhage (n = 24)	p-value
Urea (mmol/L)	3.5–10.8	47.4 (12.8–105.6)	71.2 (23.2–80.3)	0.471
Crea (µmol/L)	44–125	578 (253–1.588)	652 (424–1.200)	0.624
Plt (G/L)	150–500	225 (33–507)	181 (61–470)	0.603
PT (s)	13.8–23.2	17.5 (12.4–32.5)	19.2 (14.0–30.8)	0.436
aPTT (s)	10.0–13.2	13.8 (10.7–18.1)	16.2 (14.8–20.0)	0.053
D-dimers	<200	200 (90–2,800)	200 (100–600)	0.725
R (min)	1.8–8.6	4.2 (0.8–8.7)	4.6 (3.2–9.3)	0.686
K (min)	1.3–5.7	1.7 (0.8–3.9)	1.5 (1.0–5.1)	0.954
Alpha angel (°)	36.9–74.6	65.7 (45.1–80.0)	65.7 (31.6–75.7)	0.624
MA (mm)	42.9–67.9	67.0 (37.5–81.0)	60.0 (61.3–74.4)	0.584
Ly 30 (%)	0–8	2.0 (0.0–47.0)	2.3 (0.0–10.9)	0.928
Ly 60 (%)	0–15	3.35 (0.0–56.7)	2.9 (0.0–17.1)	0.976
G (dyn/cm^2^)	3.200–9.600	10.154 (3.000–21.316)	11.129 (7.920–14.531)	0.583

Normally distributed data are presented as mean ± standard deviation and were analyzed using the t-test. Not normally distributed data are presented as median (range) and were analyzed using the Mann–Whitney U test. aPTT=Activated partial thromboplastin time, Crea=Creatinine, G=Global clot strength, K=Kinetics, Ly30=Clot lysis after 30 min, Ly60=Clot lysis after 60 min, MA=Maximum amplitude, p-value=Significance level, Plt=Platelets, PT=Prothrombin time, R=Reaction time, RI=Reference interval

## Discussion

This study evaluated primary, secondary, and tertiary hemostasis in dogs with renal azotemia with serum creatinine values >220 μmol/L. The most common findings in these azotemic dogs were prolonged aPTT (74%), increased D-dimers (58%), decreased platelet counts (32%), hypercoagulable TEG patterns (55%), and increased clot lysis (19%).

### Coagulation times

In this study, an increase in aPTT was observed in almost 75% of the patients. Prothrombin time was significantly prolonged in dogs with AKI compared with dogs with CKD. Increased clotting times can be caused by several factors, including uremic toxins, comorbidities, loss of coagulation factors, metabolic changes caused by kidney disease, disseminated intravascular coagulation due to systemic inflammation, and endothelial damage caused by comorbidities [9, 11]. In this study, the identified causes of AKI in dogs with prolonged coagulation times were leptospirosis, grape intoxication, NSAID intoxication, and post-anesthetic AKI. Prolonged coagulation times (aPTT in 23% of the dogs, PT in 14% of the dogs) have been observed before in dogs suffering from leptospirosis [9]. Four dogs with leptospirosis were included in this study. All four had increased aPTT, and two dogs had increased PT values. Leptospirosis, as well as systemic inflammation, causes depletion of coagulation factors by decreasing the synthesis function of the liver, but also consumption of coagulation factors by ongoing hemorrhages and disseminated intravascular coagulopathy (DIC), which can lead to prolonged coagulation times, decreased platelet counts, and increased D-dimer levels [9]. In the present study, signs of DIC were observed in seven dogs with AKI, one dog with CKD, and one dog with protein-losing nephropathy. In addition, dilution due to previous fluid therapy can also increase the coagulation time, especially in oliguric patients [12].

In a previous study on dogs with CKD (9/10 IRIS stage III, 1/10 IRIS Stage IV), aPTT and PT were not different from those in healthy control dogs. These coagulation disturbances are suspected to occur in the advanced stages of the disease as well as in comorbidities. Increased D-dimer levels, as a sign of thrombolysis, were observed in 2/11 dogs. Most dogs included in this study were classified as having IRIS stage III [13]. The causes of CKD were not determined in most dogs in the present study. D-dimer levels were increased in 5/10 dogs with CKD and increased to > 2,000 ng/mL in two dogs with protein-losing nephropathy.

### Platelet count

A decreased platelet count was observed in almost 50% of the dogs with AKI and 20% of the dogs with CKD. One dog with CKD had protein-losing nephropathy. Leptospirosis was the underlying disease in three dogs with AKI and thrombocytopenia. In a previous study on dogs with leptospirosis, thrombocytopenia was observed in 60% of the dogs [9], while in another study, which included one dog with leptospirosis, platelet count was higher in 10 dogs with AKI compared to the control group [14]. About 50% of dogs included in that study were diagnosed with AoC. The chronic component of the disease may have caused a higher platelet count than that in healthy dogs.

### Viscoelastic tests

Most of the dogs included in this study had hypercoagulable TEG (55%), whereas hypocoagulable TEG was detected in 10% of the dogs. Only two previous studies have assessed platelet function using TEG in dogs with kidney diseases [13, 15].

### Hypocoagulability

Most studies have used other platelet function tests, including buccal mucosal bleeding time or platelet aggregometry, to assess hemostasis in azotemic dogs [14, 16, 17]. In these studies, the buccal mucosal bleeding time to assess hemostasis in azotemic dogs was increased up to four times, but was not associated with a decreased amount or function of von Willebrand factor [16, 17]. The study on dogs with AKI, buccal mucosal bleeding time was prolonged, with a significantly lower activated platelet aggregometry measured area under the curve and higher von Willebrand factor antigen to collagen binding activity ratio [14]. These findings explain decreased platelet function, especially in dogs with acute azotemia. Only dogs with AKI in this study showed decreased platelet function as detected by TEG.

Hypocoagulable states, detected using ROTEM, were reported in 20% of the dogs with leptospirosis [9]. Interestingly, 7/76 dogs with protein-losing nephropathy had hypocoagulable TEG [15]. These findings suggest decreased platelet function in dogs with acute azotemia. Hypocoagulability could be explained by disturbances in the alpha-granules of platelets, which decrease platelet activity in azotemic patients [11]. In addition, systemic inflammation, as observed in dogs with leptospirosis, can cause DIC, leading to the consumption of platelets and coagulation factors [9]. In the present study, three dogs with hypocoagulable TEG tracings were suffering from AKI, without evidence of leptospirosis. Two dogs also had increased fibrinolysis and 2/3 had prolonged PT times. One dog was thrombocytopenic, and D-dimer levels increased in another dog.

### Hypercoagulability

In the present study, hypercoagulable TEG profiles were observed in almost all dogs (8/10) with CKD, but only in approximately 25% of the dogs with AKI. The identified causes of azotemia in hypercoagulable dogs include protein-losing nephropathy (n = 2), hereditary kidney disease (n = 1), urinary tract infection (n = 1), grape intoxication (n = 1), post-NSAID intoxication CKD (n = 1), and leptospirosis (n = 1). Hypercoagulable TEG tracings were also reported in 40% of the dogs with leptospirosis [9], approximately 36% of the dogs with CKD stages III and IV [13], and in 89% of the dogs with protein-losing nephropathy [15]. Hypercoagulability is most likely the result of multiple factors, including the loss of proteins with anticoagulant properties, changes in the endothelium, comorbidities, or changes related to reduced GFR [13, 15]. Other proposed mechanisms of hypercoagulability in azotemic patients include increased levels of fibrinogen, coagulation factors II, XIIa, and VIIa, activated protein C complex, and thrombin-antithrombin complexes. In addition, increased platelet activity and changes in the endothelium contribute to a hypercoagulable state [11].

### Fibrinolysis

Hyperfibrinolysis was present in 5/17 dogs with AKI and 2/10 dogs with CKD, as indicated by increased Ly30 in three dogs, increased Ly30 and Ly60 in three, and increased Ly60 alone in one dog. Two of these seven dogs survived, while five did not. Two non-survivors were diagnosed with DIC. Hyperfibrinolysis was also reported in one dog with leptospirosis. In that study, increased clot lysis did not influence survival or hemorrhage [9].

### Correlations

None of the hemostatic parameters in this study correlated with creatinine levels, but PT correlated with IRIS grade and stage. It can be concluded that the severity of azotemia is not directly responsible for hemostatic changes. This is in accordance with other studies that did not report a correlation between the degree of azotemia and hemostatic imbalances [9, 15], which suggests that individual factors and the underlying disease play an important role in the development of hemostatic disorders. An experimental study found a correlation between increased buccal mucosal bleeding time and blood urea nitrogen concentration [17]. In dogs with AKI, the von Willebrand factor antigen to collagen binding activity ratio, but no other parameters, was strongly correlated with creatinine levels [14]. These findings suggest no correlation between azotemia severity and hemostatic parameters. As these studies involved only a small number of patients with a limited range of disease severity, further studies are required to assess the correlation between hemostatic parameters and disease severity.

### Survival

The surviving dogs in the present study had lower urea values and increased aPTT and D-dimer levels less often than non-surviving dogs. Survival was not associated with hypercoagulable TEG findings. In a study on dogs with leptospirosis, survival was associated with higher G-values and higher values of maximum clot firmness compared with non-survivors [9], while in the present study, 3/4 dogs with leptospirosis did not survive. All four had a G-value in the reference range and prolonged aPTT, and three had increased D-dimers levels and decreased platelet count. The results of this study suggest that increased aPTT and D-dimer levels are possible signs of DIC, and TEG results may not influence survival in azotemic dogs.

### Clinical signs of hyper- and hypocoagulability

No clinical signs of hypercoagulability or thromboembolic events were observed in this study, which is in contrast to human [5–7] and a previous veterinary study [15]. A thromboembolism incidence of 6.6% was reported previously in dogs with protein loosing nephropathy [15]. However, the diagnosis of thromboembolism can be challenging because not all thromboembolic events have observable clinical signs.

In this study, hemostatic values did not differ between dogs with and without clinical signs of bleeding. However, these were observed in 4/16 dogs with AKI, and 1/10 dogs with CKD. In a study on dogs with leptospirosis, dogs with clinical signs of hemorrhagic diathesis had a lower platelet count and higher D-dimer levels than dogs without clinical hemorrhages [9]. In one study involving 10 dogs suffering from AKI, clinical hemorrhage was observed in 30% of the dogs. These patients also exhibited decreased platelet function and impaired buccal mucosal bleeding time [14]. As hypocoagulability, DIC, and thrombocytopenia mostly occur in AKI, these dogs seem to be predisposed to clinical hemorrhage.

### Limitations

The major limitation of the present study was that the assignment of dogs to subgroups was not based on histological examination of the kidneys. In addition, the study included patients who had received drugs capable of altering platelet function, such as NSAIDs, glucocorticoids, dipyrone, and fluid therapy, before blood collection, which may have influenced the platelet count, coagulation times, and TEG values.

## Conclusion

The interactions between kidney disease and hemostasis are complex processes that are not completely understood. Although hypercoagulable TEG tracings were observed in dogs with CKD, coagulation times were prolonged in dogs with AKI. Therefore, the clinical significance of these findings remains to be determined. Further studies investigating the complex relationship between hemostasis and kidney diseases and their clinical relevance are needed.

## Authors’ Contributions

HF, VG, RD, KH, and ReD: Conceptualization. HF and ReD: Methodology, software, formal analysis, and writing-original draft preparation, investigation, and data curation. VG, RD, and KH: Validation. VG, RD, KH and ReD: Writing-review and editing and supervision. All authors have read, reviewed, and approved the final manuscript.
